# Exploring the treatment delay in the care of patients with ST-elevation myocardial infarction undergoing acute percutaneous coronary intervention: a cross-sectional study

**DOI:** 10.1186/s12913-015-0993-y

**Published:** 2015-08-21

**Authors:** Joppe Tra, Ineke van der Wulp, Martine C. de Bruijne, Cordula Wagner

**Affiliations:** Department of Public and Occupational Health, EMGO+ / VU University Medical Center, van der Boechorststraat 7, 1081 BT Amsterdam, The Netherlands; The Netherlands Institute of Health Services Research (NIVEL), Otterstraat 118, 3513 CR Utrecht, The Netherlands

## Abstract

**Background:**

A short delay between diagnosis and treatment for patients diagnosed with ST-elevation myocardial infarction (STEMI) is vital to prevent cardiac damage and mortality. The objective of this study was to explore the treatment delay and associated factors in the management of patients diagnosed with STEMI going for percutaneous coronary intervention (PCI).

**Methods:**

In a cross-sectional multicenter study, the treatment delay (time between first electrocardiogram and start of PCI procedure) of STEMI patients in seven PCI centers in the Netherlands was measured. Data were analyzed by means of multivariable generalized linear models, accounting for a non-normally distributed outcome and clustering of patients within centers.

**Results:**

In total, 1017 patient charts were included. The majority of the patients (78.7 %) were treated within the guideline recommended time target of 90 min. Overall, the median treatment delay was 64 min (interquartile range 47–82). A significantly prolonged delay was found among patients of whom their first electrocardiogram was performed at a general practitioner’s practice (+23.9 min; 95 % confidence interval 9.9–40.8) or in-hospital (+9.5 min; 95 % confidence interval 2.5–17.3), patients requiring interhospital transfer (+14.6 min; 95 % confidence interval 7.6–22.4) or presenting with acute heart failure on admission (+17.6 min; 95 % confidence interval 7.9–28.7).

**Conclusions:**

Despite a short median delay between first electrocardiogram and PCI, the time targets are occasionally exceeded for patients diagnosed with STEMI. To further improve the process of care, PCI centers should focus on improving regional STEMI care networks, involving general practitioners, emergency departments and referring hospitals.

## Background

There is a strong association between the time to reperfusion treatment and mortality for patients with ST-elevation myocardial infarction (STEMI) [1]. Every 30 min delay from symptom onset to percutaneous coronary intervention (PCI) increases patients’ risk of dying by 7.5 % [2]. In contrast to other factors that influence the likelihood of dying from STEMI, e.g. heart failure, diabetes or an anterior infarction [3], the time to PCI can be influenced immediately by health care providers. Furthermore, time to PCI can be reduced in the long run by optimizing the process of care within PCI centers and regions [4].

The importance of timely PCI treatment has been acknowledged by the European Society of Cardiology in their guidelines on the management of patients diagnosed with STEMI [5]. These guidelines recommend primary PCI over fibrinolysis as the preferred reperfusion treatment, yet only when it can be performed within 120 min after first (para) medical contact. However, treatment within 90 min from first (para) medical contact is highly preferred, or even within 60 min in case the patients’ symptoms started less than two hours ago, or they present directly to a PCI center.

Despite these recommendations, previous studies reported that a substantial number of patients are not treated within the recommended time targets, putting them at increased risk of dying from STEMI [6–8]. Certain patient groups experienced a longer treatment delay e.g. patients who are transported from a referring hospital to a PCI center (interhospital transfer) [9, 10]. Since these studies were performed, new management guidelines for STEMI were published by the European Society of Cardiology which recommend strategies on limiting delay, e.g. by building STEMI care networks consisting of PCI centers, referring hospitals and ambulance services [5].

In the Netherlands, 30 out of 91 hospitals provide PCI, serving an average population of approximately 0.6 million citizens per PCI center. Due to the good geographical accessibility of PCI centers in the Netherlands [11], PCI is the standard treatment for all patients diagnosed with STEMI without contra-indications for PCI. Once patients contact the ambulance services and they are suspected of STEMI, the goal is to directly transport them to the nearest PCI center [12]. However, patients can also contact the general practitioner or emergency department, resulting in deviating patient routes.

The process of care for patients with STEMI going for PCI is complex, and improving it is difficult [13]. By identifying patients with a prolonged treatment delay, improvements in the current strategies can be facilitated or additional strategies can be developed. In the present study the degree to which patients were treated within the European guideline recommended time targets was explored, as well as patient and admission characteristics associated with a prolonged treatment delay.

## Methods

### Study design

A cross-sectional multicenter study was conducted in the Netherlands. The study design, setting and methods have been previously described in detail elsewhere [14] and are briefly outlined below.

### Setting and population

In total, seven PCI centers in the Netherlands, selected by means of a multistage random selection procedure, participated in the present study. The number of acute PCI procedures for STEMI patients performed by the included PCI centers ranged from approximately 250 to more than 500 procedures per year, and five centers also provided thoracic surgery (Table 1).

**Table 1:** PCI center characteristics (*n* = 7)

Center nr	Type	Thoracic surgery	Nr of acute PCIs for STEMI per year^a^	Nr of patients included
1	Teaching	No	<300	127
2	Teaching	Yes	>500	112
3	Teaching	No	300–400	171
4	Academic	Yes	>500	236
5	Academic	Yes	400–500	139
6	Teaching	Yes	>500	112
7	Teaching	Yes	400–500	120

*PCI* percutaneous coronary intervention, *STEMI* ST-elevation myocardial infarction

^a^Based on data from the Dutch health care inspectorate, categorized to guarantee anonymity of the participating centers

Monthly, from each PCI center patients diagnosed with STEMI who were discharged between January 1^st^ and December 31^st^ of 2012 were preselected by means of the hospital financial system codes. The charts were screened manually to confirm a documented discharge diagnosis of STEMI. In case the discharge diagnosis was unclear or ambiguous, the chart was discussed with a cardiologist or other on-site physician working in cardiology. Charts of patients without a discharge diagnosis of STEMI, a secondary infarction (e.g. due to anemia) or with insufficient information were excluded from the study. Also, charts of patients with a documented sub-acute or old infarction (e.g. patients with an infarction of more than 12 h old, where PCI offers no clinical benefit over pharmacological treatment anymore), or ST-resolution on the electrocardiogram in combination with the absence of symptoms on admission were excluded from the study. Finally, charts of patients with a treatment delay of more than 6 h were excluded from the study, as it was unlikely that the goal was to treat these patients with PCI immediately. As a result of the eight hour shifts of the data abstractors, patients were included in the study in chronological order of discharge until the researchers were practically unable to screen additional charts on the day of the measurement.

### Data collection

Data were abstracted from patient charts by six researchers. Because the time of first (para) medical contact was not registered consistently in all PCI centers, the time of the first (pre) hospital electrocardiogram was abstracted. In case patients developed a STEMI while being hospitalized for another illness or complaint, the time of the first electrocardiogram with ST-elevation in-hospital was registered. In addition, the time of sheath insertion at the catheterization room was registered as start of the PCI procedure. As a result, the treatment delay was defined as the time between first (diagnostic) electrocardiogram and sheath insertion. Additionally, demographic characteristics, cardiac risk factors, cardiac history and admission characteristics were abstracted from patient charts.

To ensure reliable data abstraction 80 charts (6.8 %) were screened by two researchers independently and the findings were compared. Differences were discussed until consensus was reached and adapted in the original case report form accordingly. Interrater reliability was calculated by means of percentages agreement for each extracted variable and ranged between acceptable (70 %) and perfect (100 %), with exception of the variable ‘type of first (para) medical contact’ (68 %). As a result of the latter variable’s lower reliability and its unlikely influence on the delay starting from first electrocardiogram, this variable was excluded from the analyses.

### Missing data

The treatment delay was calculated by determining the difference in minutes between the first electrocardiogram and sheath insertion. The resulting values were screened and negative values were set to missing.

In total, 6.8 % of the values of all variables in the dataset were missing. The percentage of missing values per variable ranged from 0.1 % (i.e. resuscitation, age, length of stay) to 31.6 % (time from chest pain to first electrocardiogram). The variables gender, month of discharge and weekend presentation had no missing values. To explore the underlying missing data mechanism, associations between missing and non-missing values were studied and Little’s test was conducted in IBM SPSS (version 20 for Windows). The test was significant (*p* < 0.001), indicating that missing values in the dataset were not missing completely at random but either missing at random or missing not at random [15]. As a result, the missing data were imputed with multiple imputation by assuming the data were missing at random, in which the probability for a variable being missing is dependent of the value of other variables in the dataset. The data were imputed following the approach of van Buuren [16]. Prior to imputation, non-normally distributed continuous variables i.e. time from chest pain to electrocardiogram, treatment delay, and creatinine level on admission were transformed using the Box-Cox power transformation [17]. In total, 32 imputed datasets were created, following recommendations of White *et al.* [18]*.* The plausibility of the imputed data and the assumed missing data mechanism were checked by exploring the distributions of the imputed data in comparison to the original data. In these analyses it appeared that the distributions of the imputed variables were comparable to those of the original data. It was therefore assumed that the imputation resulted in plausible values. After imputation, all transformed variables were transformed back to their original units. Model estimates were calculated for each dataset and pooled using Rubin’s rules embedded in the *mice* procedure.

Data were imputed using the *mice* package in R (version 3.0.0 for Microsoft Windows) [19].

### Statistical analyses

Descriptive statistics were used to report patient and admission characteristics as well as to determine the frequency of patients who were treated within 90 and 120 min after first electrocardiogram. In addition, we investigated the frequency of being treated within 60 min for the subgroup of patients whose symptoms started less than 2 h ago or who presented directly to a PCI center. For continuous variables with an approximately normal distribution, the means with 95 % confidence intervals (CI) are described, while for continuous variables with a skewed distribution the medians with interquartile ranges (IQR) are described. The associations of patient and admission characteristics with the treatment delay were tested using generalized linear models which take into account a skewed (Gamma distributed) outcome variable. Additionally, a log link was applied because negative time values are impossible.

The goal of the study was to explore factors associated with the treatment delay. Therefore in univariate analyses, it was tested which variables were significantly (*p* < 0.05) associated with treatment delay. Explanatory variables with a significant effect on treatment delay were entered into a multivariable generalized linear model. To correct for clustering of patients in PCI centers, the variable ‘PCI center’ was added as a covariate in all analyses. Moreover, the process of care might differ between PCI centers, and therefore associations between explanatory variables and the outcome variable might differ. To correct for these differences, interaction terms between the explanatory variables and PCI center were tested and added to the model in case they significantly improved the model fit.

Additionally, because collinearity can lead to unjustified exclusion of explanatory variables, all variables not significantly associated in the univariate analyses were added to the multivariable model one by one. Variables with a significant improvement of the model fit were added to the final multivariable model. The results of the final multivariable model were interpreted as the minimal delay in minutes per variable, holding all other variables constant. As a result of the underlying missing data pattern of missing at random, all reported results were based on analyses of the imputed data.

### Ethical approval

The study protocol was approved by the medical ethical review committee of the VU University medical center. Anonymity of patients and PCI centers was protected by coding patient and center characteristics i.e. no names or addresses of patients or centers, or patient identification numbers were recorded. Data were stored on a password protected network of the EMGO+ / VU University medical center. All chart abstractors signed confidentiality agreements, and the study was registered with the Dutch Data Protection Agency.

## Results

In total, a sample of 1170 charts of patients with a confirmed discharge diagnosis of STEMI was selected. Of these charts, 150 (12.8 %) patients received pharmacological treatment or non-acute PCI, and 3 (0.3 %) had a treatment delay of more than six hours. These patient charts were subsequently excluded from the study, leaving 1017 charts for further analyses. The number of included charts per PCI center ranged from 112 to 236.

The mean age of patients presenting with STEMI and going for acute PCI was 62.5 years (95 % confidence interval: 61.3–63.7) (Table 3). The median time between symptom onset and first electrocardiogram was 81 min (interquartile range: 20–142) (Table 4).

### Treatment within guideline recommended times

The treatment times and ischemic times of the observed data are presented in Table 2. After imputation of the missing values, the median time from first electrocardiogram to PCI was 64 min (interquartile range: 47–82). PCI centers were able to treat 800 (78.7 %) patients within 90 min from first electrocardiogram with PCI, and 918 (90.3 %) patients within 120 min. Of the patients who presented within two hours of symptom onset or who presented directly to a PCI center (*n* = 700), 312 (44.6 %) were treated within 60 min as recommended by the guidelines.

**Table 2:** Treatment delay and ischemic time in various patient groups based on observed data

Characteristic	Number	Missing (%)	Treatment delay^a^	Missing (%)	Ischemic time^b^
All patients	1017	139 (13.7 %)	64 (51–84)	330 (32.4 %)	148 (105–266)
Interhospital transfer for PCI	1009				
- yes	199	25 (21.6 %)	77 (58–110)	76 (38.2 %)	173 (118–274)
- no	810	110 (13.6 %)	62.0 (49–79)	248 (30.6 %)	145 (101–219)
First ECG	874				
- General practitioner	25	2 (8.0 %)	86 (68–113)	4 (16.0 %)	199 (161–341)
- Ambulance	707	55 (7.8 %)	62 (49–78)	184 (26.0 %)	141 (100–215)
- Hospital	142	10 (7.0 %)	77 (60–111)	49 (34.5 %)	189 (119–324)

*PCI* percutaneous coronary intervention, *ECG* electrocardiogram

^a^Treatment delay was defined in minutes as ranging from first electrocardiogram with ST-elevation to start of the percutaneous coronary intervention and is not corrected for the effects of other independent variables

^b^Ischemic time was defined in minutes as ranging from symptom onset to start of the percutaneous coronary intervention

### Factors associated with treatment delay

In univariate analyses, of the patient characteristics, a prior myocardial infarction (*p* = 0.046) and prior use of anticoagulants (within the last seven days) (*p* = 0.007) were significantly associated with treatment delay (Table 3).

**Table 3:** Original and imputed patient characteristics and associations with treatment delay in univariate analyses (*n* = 1017)

Variable	Missing (%)	Original value	Imputed value	*p*-value*
Age (mean (SD) years)	1 (0.1 %)	62.5 (12.5)	62.5 (12.5)	0.16
Female	0 (0 %)	271 (26.6 %)	271 (26.6 %)	0.63
Diabetes Mellitus	78 (7.7 %)	124 (13.2 %)	136 (13.4 %)	0.91
Hypertension	84 (8.3 %)	375 (40.2 %)	406 (39.9 %)	0.52
Hyperlipidemia^a^	89 (8.8 %)	303 (32.7 %)	335 (32.9 %)	0.35
Obesity (BMI > 30 kg/m^2^)	87 (8.6 %)	80 (8.6 %)	90 (8.8 %)	0.54
(Ex-) smoker	88 (8.7 %)	526 (56.6 %)	563 (55.4 %)	0.38
Prior myocardial infarction	102 (10.0 %)	123 (13.4 %)	144 (14.2 %)	**0.046**
Prior PCI	102 (10.0 %)	132 (14.4 %)	158 (15.5 %)	0.06
Prior CABG	103 (10.1 %)	21 (2.3 %)	30 (2.9 %)	0.55
Prior use of anticoagulants (≤7 days)	86 (8.5 %)	183 (19.7 %)	205 (20.2 %)	**0.007**

Significant results are highlighted in **bold**

*SD* standard deviation, *BMI* body mass index, *kg/m2* kg per square meter, *PCI* percutaneous coronary intervention, *CABG* coronary artery bypass graft

*P-values are calculated using the Wald statistics, comparing a generalized linear model with and without the imputed variable, corrected for clustering of patients in PCI centers

^a^Hyperlipidemia is defined as described in patient's history or statin use before admission

Moreover, of the admission characteristics, the location where the first electrocardiogram was made (*p* < 0.001), interhospital transfer for PCI (*p* < 0.001), acute heart failure on admission (*p* < 0.001), creatinine level on admission (*p* = 0.03) and a stenosis in the left anterior descending coronary artery (*p* = 0.03) were significantly associated with treatment delay (Table 4).

**Table 4:** Original and imputed admission characteristics and associations with treatment delay in univariate analyses (*n* = 1017)

Variable	Missing (%)	Value	Imputed value	*p*-value*
Time from chest pain to ECG (median (IQR) minutes)	321 (31.6 %)	78 (41–148)	81 (20–142)	0.12
Treatment delay (median (IQR) minutes from ECG to PCI)	139 (13.7 %)	64 (51–84)	64 (47–82)	N/A
First ECG	143 (14.1 %)			**<0.001**
- General practitioner		25 (2.9 %)	34 (3.3 %)	
- Ambulance		707 (80.9 %)	809 (79.6 %)	
- Hospital		142 (16.2 %)	174 (17.1 %)	
Weekend presentation	0 (0 %)	291 (28.6 %)	291 (28.6 %)	0.92
Weekday evening presentation	152 (14.9 %)	385 (44.5 %)	450 (44.2 %)	0.15
Month of discharge	0 (0 %)	N/A	N/A	0.72
Interhospital transfer for PCI	8 (0.8 %)	199 (19.7 %)	200 (19.7 %)	**<0.001**
Systolic blood pressure on admission (mean (SD) mmHg)	45 (4.4 %)	130 (26)	130 (27)	0.82
Heart rate on admission (mean (SD) beats/min)	161 (15.8 %)	75 (18)	75 (18)	0.48
Resuscitation	1 (0.1 %)	99 (9.7 %)	99 (9.7 %)	0.31
Acute heart failure on admission	54 (5.3 %)	47 (4.9 %)	58 (5.7 %)	**<0.001**
Cardiogenic shock on admission	71 (7.0 %)	37 (3.9 %)	46 (4.5 %)	0.06
Creatinine level on admission (median (IQR) mmol/L)	112 (11.0 %)	78 (66–91)	77.8 (65.0–90.6)	**0.03**
Nr of diseased vessels	22 (2.2 %)			0.56
- One		555 (55.8 %)	566 (55.7 %)	
- Two		264 (26.5 %)	269 (26.4 %)	
- Three		176 (17.7 %)	182 (17.9 %)	
Location stenoses^a^				
- Left main	7 (0.7 %)	27 (2.7 %)	27 (2.7 %)	0.20
- Right coronary	6 (0.6 %)	565 (55.9 %)	569 (55.9 %)	0.10
- Left anterior descending	5 (0.5 %)	648 (64.0 %)	652 (64.1 %)	**0.03**
- Circumflex	7 (0.7 %)	400 (39.6 %)	405 (39.8 %)	0.06

Significant results are highlighted in **bold**

*ECG* electrocardiogram, *IQR* interquartile range, *PCI* percutaneous coronary intervention, *N/A* not applicable, *SD* standard deviation, *mmHg* millimeter of mercury, *mmol/L* millimoles per litre

*P-values are calculated using the Wald statistics, comparing a generalized linear model with and without the imputed variable, corrected for clustering of patients in PCI centers

^a^Only stenoses ≥50 %. A single patient can have more than one stenosis

When looking at differences in delaying factors per PCI center, no interactions of PCI center with patient or admission characteristics were significantly associated with treatment delay.

Variables with a significant association with treatment delay in univariate models were added to a multivariable generalized linear model. One variable with a non-significant association in univariate analyses improved the multivariable model fit (stenosis in the circumflex artery, *p* = 0.03) and was added to the multivariable model. No interactions significantly improved the multivariable model fit. In the final multivariable model, patients of whom their first electrocardiogram was performed at a general practitioner (+23.9 min; 95 % CI 9.9–40.8) or hospital (+9.5 min; 95 % CI 2.5–17.3) had an additional treatment delay compared to patients of whom their first electrocardiogram was made in the ambulance (Table 5). Moreover, patients who required interhospital transfer (+14.6 min; 95 % CI 7.6–22.4), with acute heart failure on admission (+17.6 min; 95 % CI 7.9–28.7) or with a stenosis in the circumflex artery (+4.3 min; 95 % CI 0.4–8.6) had a significantly longer treatment delay.

**Table 5:** Associations of patient and admission characteristics with treatment delay in the multivariable analysis

Variable	% increase in treatment delay (95 % CI)		Minimal increase in treatment delay in minutes (95 % CI)^a^		*p*-value
Intercept	N/A		59.8	(51.5;69.4)	N/A
Prior myocardial infarction	3.0 %	(−7.6;14.9)	1.8	(−4.5;8.9)	0.59
Prior use of anticoagulants (≤7 days)	6.7 %	(−2.3;16.5)	4.0	(−1.4;9.9)	0.15
First ECG at the general practitioner	40.0 %	(16.5;68.2)	23.9	(9.9;40.8)	**<0.001**
First ECG in the hospital	15.9 %	(4.1;28.9)	9.5	(2.5;17.3)	**0.007**
Interhospital transfer for PCI	24.4 %	(12.7;37.4)	14.6	(7.6;22.4)	**<0.001**
Acute heart failure on admission	29.5 %	(13.2;48.0)	17.6	(7.9;28.7)	**<0.001**
Creatinine level on admission (per mmol/L)	0.1 %	(−0.04;0.2)	0.06	(−0.02;0.14)	0.16
Stenosis in left anterior descending	4.1 %	(−2.2;10.9)	2.5	(−1.3;6.5)	0.21
Stenosis in circumflex artery	7.3 %	(0.6;14.4)	4.3	(0.4;8.6)	**0.03**

Significant results are highlighted in **bold**

*N/A* not applicable, *CI* confidence interval, *PCI* percutaneous coronary intervention, *ECG* electrocardiogram

^a^As a result of the generalized linear model with a gamma distributed outcome and a log link, combining the effects of independent variables should be performed with caution due to multiplicative effects (and not additive) effects

## Discussion

Treating STEMI patients with primary PCI within the guideline recommended time frames is complex, and strategies to achieve them have been published in international guidelines in recent years. In this study, the treatment delay of patients with STEMI and associated factors were explored. Despite a median treatment delay of 64 min, the time target of 90 min was exceeded in 21.3 % of the patients. In an attempt to explain these findings, factors associated with a prolonged treatment delay were identified. Patients of whom an electrocardiogram was made at a general practitioner or hospital, patients requiring interhospital transfer for PCI, patients with acute heart failure or with a stenosis in the circumflex artery were more likely to experience a prolonged treatment delay. These patients have an increased but potentially preventable risk of cardiac damage and dying.

Several studies have investigated treatment delay previously, and found a longer median treatment delay than in this study [9, 10, 20]. This finding may be explained by the use of a different definition of delay, e.g. from first (para) medical contact to PCI instead of first electrocardiogram to PCI. It may also be explained by the relatively high number of PCI centers in the Netherlands distributed over the country, resulting in good geographical accessibility.

The delaying factors identified in this study are comparable to other studies, in which patients who were transferred between hospitals had a prolonged treatment delay [9, 10, 21]. Also, a prolonged treatment delay and higher mortality were found for patients who first contacted a general practitioner instead of direct contact with emergency medical services or the hospital [22]. However, in the present study, patients of whom their first electrocardiogram was made at a hospital also had a prolonged delay than patients who contacted the emergency medical services directly. These findings confirm that contacting the ambulance services when experiencing cardiac complaints is of vital importance for patients with a potential STEMI in limiting treatment delay. While transporting a patient directly to a PCI center, ambulance services can contact the PCI center and initiate catheterization room activation [23]. This supports the European Society of Cardiology recommendation that the general population should be encouraged to contact the ambulance services instead of the general practitioner or hospital when experiencing symptoms of STEMI [5]. However, influencing the behavior of potential STEMI patients is difficult [24, 25]. Therefore, new interventions to reduce patient delay are required, but until then improvement efforts should focus on reducing system delay (time from first (para) medical contact to wire passage).

Additionally, there has been a call to form STEMI care networks with which providers of STEMI care can initiate interventions to speed up the process of care. In the Netherlands, this initiative has resulted in a national program to improve organization of STEMI care per region [26]. For these initiatives, it is recommended to involve all relevant stakeholders i.e. PCI centers, referring hospitals, emergency departments and general practitioners within the network region. Previous improvement studies which excluded patients who were transferred between hospitals found no significant survival benefit of reducing the door-to-balloon time by 16 min [27]. In this study, we have shown that interhospital transfer was one of the strongest predictor for a prolonged treatment delay. These patients therefore potentially benefit the most from reducing the treatment delay, which might result in improvements in survival. Consequently, in future studies it is important to include all patients undergoing primary PCI.

The finding that patients with acute heart failure were more likely to experience a prolonged treatment delay than patients without acute heart failure may be explained by the fact that they require more elaborate stabilization in the acute care phase. It is uncertain whether this delay can be reduced without compromising the safety of the patient, and therefore a longer delay might have to be accepted.

Also patients presenting with a stenosis in the circumflex artery experienced a significantly prolonged treatment delay. A stenosis in the circumflex artery might indicate a lateral or posterior infarction. These patients tend to present with limited electrocardiogram abnormalities [28], which hampers rapid diagnosis. Use of a posterior electrocardiogram in the ambulance may speed up diagnosis and reduce the treatment delay for these patients. However, the difficulty of interpreting a posterior electrocardiogram might result in unnecessary activations of the catheterization room, and continuous education for paramedics in the regional STEMI care networks might be required.

### Limitations

Some limitations of this study need to be taken into account when interpreting the results. First, the time from first (para) medical contact to wire passage, as specified in the European Society of Cardiology guidelines, was not registered in all participating PCI centers. Therefore, the time from first electrocardiogram (diagnosis) to sheath insertion was used to determine the treatment delay. This definition is only part of the time to reperfusion as described in cardiac guidelines [5, 6], who differentiate between patient delay (symptom onset to first (para) medical contact) and system delay (first (para) medical contact to wire passage). As a consequence, the results of this study underestimate the treatment delay of STEMI patients in the Netherlands compared to other studies [29].

Second, the treatment times registered in the participating PCI centers were not primarily registered for scientific research. This could potentially compromise the validity of the registered times, which would have affected the reliability of the study findings. However, we used automatically registered times as much as possible, i.e. times registered on an electrocardiogram or in the catheterization room system, and checked the plausibility of the calculated treatment delay. Also, the equivalent time variables were used by PCI centers to report quality indicators to the national government and were therefore also used to make policy decisions.

Third, the PCI center in which a patients was treated was only used as a statistical correction in this study because of size differences between the regions in which centers were located. Consequently, in case of significant differences in the treatment delay between centers, it would be impossible to differentiate between differences caused by regional size or by differences in health care organization.

Fourth, in this study it was not possible to identify the correlation between treatment delay and hospital mortality. In the Netherlands, it is common practice to transport the patient from the PCI center to their local hospital shortly after the PCI procedure for follow-up care. Due to Dutch privacy regulations, no information of patients could be linked for research purposes between hospitals. As a result, no information about hospital mortality could be obtained.

Finally, chart review is a data collection method with a high risk of missing values. However, it would be inappropriate to delete all cases with missing values as this can introduce bias in the study population. Therefore extensive methods were used in this study to account for these missing values.

## Conclusions

Despite a median treatment delay within guideline recommendations, patients diagnosed with STEMI who required PCI treatment were occasionally not treated on time as recommended in the European guidelines. A first electrocardiogram made at the general practitioner or hospital, and interhospital transfer resulted in a prolonged treatment delay for these patients. To further improve the process of care, efforts should focus on strengthening STEMI care networks, incorporating all relevant stakeholders in a region including general practitioners, referring hospitals and emergency departments.

## Abbreviations

STEMI: ST-elevation myocardial infarction
PCI: Percutaneous coronary intervention
CI: Confidence interval
IQR: Interquartile range
